# 

**Published:** 1902-03

**Authors:** 


### PERSONALS.

Dr. E. J. Creely and wife, of San Francisco, Cal., made an eastern trip to the Atlantic coast in January and February. While in Philadelphia Dr. Creely paid a visit to the office of the JOURNAL.

Dr. Leonard Pearson, State Veterinarian of Pennsylvania, spoke in the four States of New York, New Jersey, Delaware, and Pennsylvania during the month of February on the subject of tuberculosis.

The position of Assistant City Veterinarian of Pittsburg has been created, and the salary fixed at fifteen hundred dollars a year. Dr. Henry Mayer has been appointed to fill the position. Dr. Mayer is a graduate of the Veterinary Department, University of Pennsylvania, class of 1900.

Dr. Vaughan Lloyd, of Richmond, Va., met with a painful accident in December through a fractured leg, but is again in practice.

Dr. J. A. Atkinson, of Goodwill Hill, Pa., has met with a serious misfortune in the burning of his home, instruments, books, and considerable money, none of which was saved out of the conflagration.

Dr. B. F. Minnick, of Columbia, Pa., is an ardent admirer of the trotting-bred horse, and has owned and maintained in the stud several standard bred stallions. He is the owner at present of an "Allerton" stud colt and a noted "Wilkes" mare.

Dr. C. E. Hyle, of York, Pa., was arrested in February for violation of the laws regulating the practice of veterinary science. Secretary Hoskins was the prosecutor.

Dr. Leonard Pearson, of Philadelphia, Pa., was a visitor to the "Bay State" in February, spending some four or five days.

Prof. Virchow, the eminent bacteriologist, was seriously ill during the month of February, but is reported convalescent.

Drs. N. Rectenwald and W. E. Wight, of Pittsburg, Pa., were in attendance at the Ohio State Veterinary meeting at Columbus in January.

President Ellis, of the Alumni Association of the American Veterinary College, was present at the banquet of the general Alumni of the University of New York.

Dr. J. C. Kingan, of Pittsburg, Pa., has opened a very commodious veterinary infirmary in the Bloomfield district of the "Smoky City."

Dr. J. O. George, of Camden, N. J., has opened his newly-built veterinary hospital under auspicious circumstances. An equine ambulance is part of his equipment.

Dr. H. P. Eves, of Wilmington, Del., recently purchased a fine veterinary ambulance from Dr. C. W. Boyd, of Allegheny, Pa.

Dr. N. Rectenwald, of Pittsburg, Pa., is very popular among the Hebrew residents of the State of Allegheny and is fast learning to parse the feminine of He-brew as "She-brew."

Dr. Edward Martin, M. R. C. V. S., has succeeded Dr. P. K. Jones at the Vancroft Stock Farm, Wellsburg, W. Va.

Dr. Horace G. Black, of Atlantic City, N. J., has returned to his former location at Wilkesbarre, where he will resume his practice among former patrons.

